# Preface to the 4th MDS Video Challenge Case Supplement for *Movement Disorders Clinical Practice*

**DOI:** 10.1002/mdc3.13832

**Published:** 2023-08-24

**Authors:** Michele Matarazzo, Christos Ganos

**Affiliations:** ^1^ Centro Integral de Neurociencias AC (CINAC) Hospital Universitario HM Puerta del Sur, Fundación de Investigación HM Hospitales Madrid Spain; ^2^ Department of Neurology Charité University Hospital Berlin Germany

**Keywords:** MDS video challenge, movement disorders, education, neurology

The MDS Video Challenge format is a central tradition of the annual International Congress of Parkinson's Disease and Movement Disorders®. This special issue documents the cases presented at the 2022 MDS Video Challenge that took place in Madrid, Spain along with the respective videos and discussions with key learning points. These cases were shortlisted from a total of 83 submissions (34 from the Asian and Oceanian section, 22 from the European section, 21 from the Pan American section, and 6 from the African section) highlighting challenging phenomenological and etiological associations. The masters of ceremony for 2022 were Drs. Anthony E. Lang and Kailash P. Bhatia, and the panel of discussants comprised Drs. Myriam Carecchio (Italy), Rob De Bie (The Netherlands), Abhimanyu Mahajan (United States), Michele Matarazzo (Spain), and Meenakshisundaram Umairorubahan (India).

This special issue includes 17 submissions, coming from 13 different countries, namely Australia, Brazil, China, India, Italy, Malaysia, Mexico, Spain, Switzerland, Thailand, Turkey, the United Kingdom, and the United States. This is a true explosion of diversity and global participation that is guided by the quality and educational merit of the cases. Indeed, the long‐standing tradition of the MDS Video Challenge continuingly succeeds in fostering a growing international culture of exquisite analytical skills and differential diagnostic thinking, and propels active engagement in clinically motivated translational science. The presented cases cover a very broad spectrum of movement disorders and etiologies, including neurogenetic, infectious, drug‐induced, metabolic, tumor‐related, and autoimmune causes.^1^, ^2^, ^3^, ^4^, ^5^, ^6^, ^7^, ^8^, ^9^, ^10^, ^11^, ^12^, ^13^, ^14^, ^15^, ^16^, ^17^ Importantly, they provide practical take‐home messages and clues to diagnosis, as means to optimize the level of care for our patients.

During the process of putting together this supplement, one striking point became apparent: the imperative of revisiting a given diagnosis. Indeed, several cases here were conclusively diagnosed after several follow‐up examinations owing, for example, to atypical or early presentations that only later evolved to full‐blown and well‐recognized clinical phenotypes, or the discovery of new, previously unknown, etiologies. This highlights the need to thoughtfully re‐evaluate cases considering new developments in our fast‐galloping field. This also includes cases that may have received a functional movement disorder diagnosis, specifically when positive clinical diagnostic signs are absent.^5^, ^11^ Although we acknowledge the increasing complexity between phenotypic and etiologic associations in light of an ever‐expanding number of conditions, as for example, neurogenetic and autoimmune, we see this as a unique opportunity for growth in an era of exciting diagnostic and treatment developments. The cases provided here, but also in the previous supplements over the years (Volume 7, Issue S3; Volume 8, Issue S1; Volume 9, Issue 2), decisively enrich our clinical knowledge and skills and aid our therapeutic decisions. They also stand as a remarkable demonstration of the unwavering dedication exhibited by the MDS Video Challenge hosts, the case reviewers, the Movement Disorder Congress team, the invited panels of expert clinicians, and, of course, all members of our society who actively contribute to this truly unparalleled collaborative international endeavor. Most importantly, the MDS Video Challenge is a testament to the selflessness and strong will of people who seek care in neurology and their families, who together with their movement disorder health professionals synergistically advance education and medical care. We, the editors of this supplement, remain indebted to all of you and wholeheartedly encourage you to continue nurturing this invaluable tradition over the years to come.

## Author Roles

1.Research project: A. Conception, B. Organization, C. Execution; 2. Statistical Analysis: A. Design, B. Execution, C. Review and Critique; 3. Manuscript Preparation: A. Writing of the first draft, B. Review and Critique

MM, CG: 3A, 3B.

## Disclosures

**Funding Sources and Conflicts of Interest**: No funding was received for this work, and the authors have no conflicts of interest to declare.

**Financial Disclosures for the Previous 12 Months**: M.M. has received research funding from The Michael J. Fox Foundation and lecture honoraria from Zambon and Palex. C.G. receives scientific research support from the VolkswagenStiftung (Freigeist) and lecture honoraria from the Movement Disorder Society.

## References

[1] Monfrini E et al. Chorea‐acanthocytosis presenting with parkinsonism‐dystonia without chorea. Mov Disord Clin Pract 2023;10(S3):S32–S34. 10.1002/mdc3.13771 PMC10448615 37636221

[2] Mohammad S et al. Oculomotor apraxia as an early presenting sign of juvenile‐onset Huntington's disease. Mov Disord Clin Pract 2023;10(S3):S12–S14. 10.1002/mdc3.13775 PMC10448626 37636223

[3] Mawardi AS et al. Myoclonus‐ataxia syndrome associated with Hyperprolinemia type I. Mov Disord Clin Pract 2023;10(S3):S38–S40. 10.1002/mdc3.13780 PMC10448612 37636236

[4] Chakraborty R et al. Jaw opening Myoclonus in Subacute Sclerosing Panencephalitis. Mov Disord Clin Pract 2023;10(S3):S63–S65. 10.1002/mdc3.13782 PMC10448621 37636229

[5] González‐Herrero B et al. Oral squamous cell carcinoma presenting as a stiff tongue. Mov Disord Clin Pract 2023;10(S3):S58–S60. 10.1002/mdc3.13769 PMC10448623 37636222

[6] Millar‐Vernetti P et al. Meropenem‐induced facial myoclonus. Mov Disord Clin Pract 2023;10(S3):S21–S23. 10.1002/mdc3.13777 PMC10448627 37636233

[7] Hull M et al. Recurrent nocturnal tongue biting in a case of hereditary Geniospasm. Mov Disord Clin Pract 2023;10(S3):S29–S31. 10.1002/mdc3.13770 PMC10448609 37636234

[8] Williams L et al. Seeing what is not there: revisiting a diagnostic conundrum in the clinic. Mov Disord Clin Pract 2023;10(S3):S54–S57. 10.1002/mdc3.13767 PMC10448618 37636225

[9] Gültekin‐Zaim ÖB et al. Cockayne syndrome type 3 with dystonia‐ataxia and clicking blinks. Mov Disord Clin Pract 2023;10(S3):S48–S50. 10.1002/mdc3.13778 PMC10448610 37636235

[10] Chavira‐Hernández G et al. Ataxia due to a COQ8A novel variant in primary coenzyme Q10 deficiency. Mov Disord Clin Pract 2023;10(S3):S41–S44. 10.1002/mdc3.13781 PMC10448619 37636224

[11] Bhowmick S et al. Unmistakable truncal dystonia mistaken as psychogenic: a case report of VAC14‐related neurodegeneration. Mov Disord Clin Pract 2023;10(S3):S15–S20. 10.1002/mdc3.13776 PMC10448624 37636228

[12] Cassou E et al. A teenager with benign hereditary chorea and selective tooth agenesis type 3. Mov Disord Clin Pract 2023;10(S3):S35–S37. 10.1002/mdc3.13779 PMC10448611 37636230

[13] Ardila‐Jurado E et al. Tongue Myorhythmia and palatal tremor as the main clinical manifestation in anti‐IgLON5 disease. Mov Disord Clin Pract 2023;10(S3):S61–S62. 10.1002/mdc3.13768 PMC10448617 37636231

[14] Heiser H et al. Generalized dystonia due to KMT2B mutation in a patient with a previous diagnosis of Russell silver syndrome. Mov Disord Clin Pract 2023;10(S3):S51–S53. 10.1002/mdc3.13794 PMC10448616 37636227

[15] Sringean J et al. Immune checkpoint inhibitor‐associated striatal encephalitis presenting with subacute progressive parkinsonism. Mov Disord Clin Pract 2023;10(S3):S7–S11. 10.1002/mdc3.13800 PMC10448625 37636220

[16] Balint B et al. Parkinsonism due to coxsackie B virus infection – case report and literature review. Mov Disord Clin Pract 2023;10(S3):S24–S28. 10.1002/mdc3.13773 PMC10448620 37636219

[17] Chen X et al. Multiple system atrophy‐like phenotype accompanied by prominent weight loss and fatigue. Mov Disord Clin Pract 2023;10(S3):S45–S47. 10.1002/mdc3.13802 PMC10448613 37636226

